# Vibrating Mesh Nebulizer (A-VMN) Performance During Low-Flow Nasal Oxygen Therapy in Neonates

**DOI:** 10.3390/pharmaceutics18070866

**Published:** 2026-07-16

**Authors:** Rachel Burke, Mary Joyce, Elena Fernández Fernández, Brendan D. Higgins, Ronan MacLoughlin

**Affiliations:** 1 Medical Affairs, Aerogen Ltd., Galway Business Park, Dangan, H91 HE94 Galway, Ireland; rburke@aerogen.com (R.B.); efernandez@aerogen.com (E.F.F.); 2 Research and Development, Science and Emerging Technologies, Aerogen Ltd., Galway Business Park, Dangan, H91 HE94 Galway, Ireland; mjoyce@aerogen.com; 3 Discipline of Physiology, School of Pharmacy and Medical Sciences, University of Galway, University Road, H91 W5P7 Galway, Ireland; brendan.higgins@universityofgalway.ie; 4 School of Pharmacy and Biomolecular Sciences, Royal College of Surgeons in Ireland (RCSI), D02 YN77 Dublin, Ireland; 5 School of Pharmacy and Pharmaceutical Sciences, Trinity College, D02 PN40 Dublin, Ireland

**Keywords:** aerosol, neonate, oxygen inhalation therapy, oxygen concentrator, vibrating mesh nebulizer

## Abstract

**Background**: Supplemental oxygen and aerosol therapy may be used simultaneously to treat neonates suffering from hypoxemia caused by respiratory diseases. Due to the cost and lack of availability of oxygen cylinders in some countries, oxygen concentrators are a reported substitute. We assessed whether an oxygen concentrator compared to low-flow oxygen therapy impacts neonatal aerosol drug delivery. **Methods**: A vibrating mesh nebulizer (A-VMN; Aerogen Solo) was used to aerosolize a 500 µg dose of salbutamol. The aerosol was delivered via a nasal cannula to a neonate head model in combination with oxygen concentrator at gas flow rates of 0.2, 1.0, and 5.0 L per minute (LPM), and low-flow oxygen therapy at gas flow rates of 1.0, 4.0, and 5.0 LPM. Emitted and tracheal doses were recorded. The impact of A-VMN operation and refill on circuit pressure in both systems was also measured. **Results**: The oxygen concentrator delivered a higher emitted dose than the low-flow system, the largest emitted dose (%) being 20.58 ± 0.50% and 14.69 ± 0.89%, respectively, at 1.0 LPM, *p* = 0.018. At 5.0 LPM, the tracheal dose was 11.01 ± 0.29% for the oxygen concentrator compared to 9.66 ± 1.53% for low-flow oxygen therapy, *p* = 0.073. Refill and operation of the A-VMN did not impact the circuit pressure in either system. **Conclusions**: This study shows that the system used to provide concurrent aerosol and supplemental oxygen therapy has a significant impact on the quantity of nebulized drug delivered to patients.

## 1. Introduction

Respiratory distress in newborns presents with signs of increased breathing work, including nasal flaring and tachypnea [1]. Oxygen therapy in neonates and pediatrics is commonly used to treat those in respiratory distress caused by asthma, bronchiolitis, wheezing, or a respiratory infection such as pneumonia [2].

Low-flow nasal oxygen (LFNO) therapy delivers medical gas via a nasal cannula and is among the most commonly used methods to deliver oxygen to infants and pediatrics [3]. The World Health Organization (WHO) considers low-flow rates to be those < 4 L per minute (LPM) for pediatric patients [4]. If LFNO therapy is unsuccessful, high-flow nasal oxygen (HFNO) therapy can be used as an alternative treatment method. HFNO, typically 2–30 LPM adjusted to patient weight, has been widely established for respiratory distress, reducing the need for intubation in both adults and preterm infants, and can be used after failure of initial LFNO therapy [2,5,6,7]. Nasal oxygen therapy in general is of particular use in infant and pediatric patient populations as they are predominantly nasal breathers [8].

Both HFNO and LFNO require an oxygen source, typically compressed gas cylinders, which are heavy, costly, and have limited availability in low-income countries [9]. One method of oxygen supplementation that can overcome these barriers is the use of an oxygen concentrator. Oxygen concentrators are usually portable, reliable, and lower cost in comparison to cylinders, and can be used to deliver LFNO therapy in the home environment [3,10]. However, disadvantages include the need for electrical power and unsuitability for patients requiring high oxygen flow rates. Recent systematic evidence has documented persistent supply–demand gaps for oxygen concentrators in resource-limited settings, further highlighting the need to characterize aerosol drug delivery performance using these devices [9].

During oxygen therapy, patients may also benefit from concurrent aerosol drug delivery, making this an attractive treatment option [11,12,13,14,15]. One survey of pediatric clinical practice suggested that concurrent aerosol therapy was delivered by 75% of respondents during HFNO, with 77% of these respondents delivering aerosol via vibrating mesh nebulizer. During aerosol therapy, 13% of respondents decreased HFNO flow, while 23% removed patients from HFNO [16]. While drug deposition via nasal cannula can be low in neonates, it has been shown to be within the potential therapeutic range for certain drugs [15,17].

To date, aerosol drug delivery efficiency during oxygen concentrator use has not been described. This in vitro study assessed aerosol drug delivery performance using an A-VMN with an oxygen concentrator in a simulated 15-day-old neonatal patient, compared against LFNO across clinically relevant gas flow rates. We hypothesized that the absence of heated humidification in the oxygen concentrator circuit would result in greater emitted and tracheal drug doses compared to LFNO at matched flow rates.

## 2. Materials and Methods

### 2.1. Nebulizer System

The Aerogen Solo vibrating mesh nebulizer (A-VMN mesh; Aerogen Pro-X1 Controller (Aerogen Ltd., Galway, Ireland)) was used throughout. The volumetric mean diameter (VMD) of aerosol droplets produced by the nebulizer was 4.38 ± 0.01 µm with an aerosol output rate of 0.47 ± 0.01 mL/min, when determined by laser diffraction as previously described [18].

### 2.2. Aerosol Delivery Characterization

**Figure 1:**
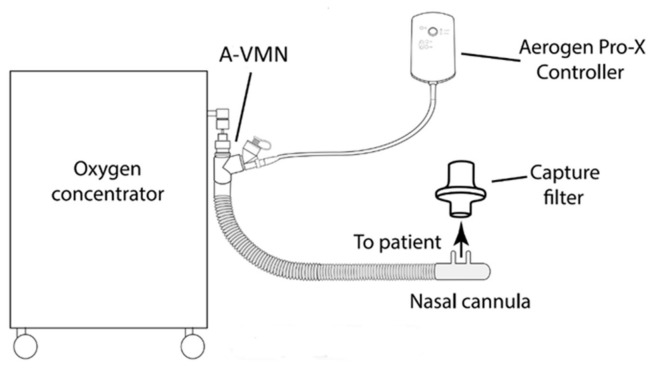
Experimental setup used to determine the emitted dose using the A-VMN with an oxygen concentrator.

**Figure 2:**
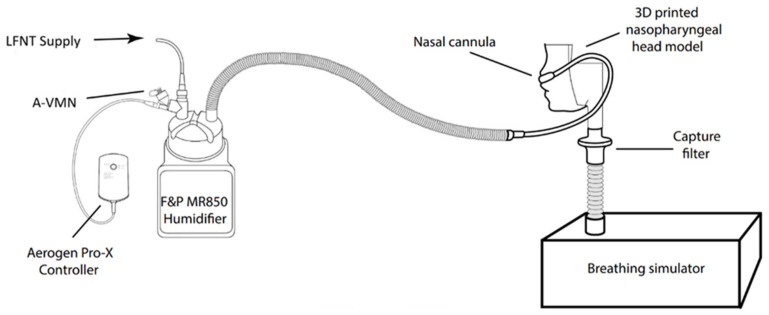
Experimental setup used to determine the tracheal dose using the A-VMN with low-flow oxygen therapy.

For both LFNO and oxygen concentrator setups, the emitted and tracheal doses, expressed as a percentage of the nominal dose, were quantified. The quantity of drug rainout in the T-piece was also quantified. The emitted dose measures equipment or system compatibility with an aerosol generator and their effect on aerosol transport through that system, independent of the patient [19]. Tracheal dose represents the dose available for inhalation at the end of the trachea of the head model. Schematic illustrations of the experimental setup are provided in Figure 1 and Figure 2.

The A-VMN was attached using a 22 mm T-piece (Aerogen Ltd., Galway, Ireland) directly to the oxygen concentrator (Oxy 6000, BITMOS Medizintechnik, Bochum, Germany) and, for LFNO, at the dry side of a humidifier system (MR850, Fisher & Paykel, Auckland, New Zealand). A 500 µg dose of aerosolized salbutamol (2.5 mg/2.5 mL, TEVA Pharmaceuticals, Dublin, Ireland) was delivered through a nasal cannula (BC2745-20, Infant nasal cannula, Fisher & Paykel, Auckland, New Zealand). The oxygen concentrator was assessed at gas flow rates of 0.2, 1.0, and 5.0 LPM and the LFNO was assessed at gas flow rates of 1.0, 4.0, and 5.0 LPM. All testing was conducted under controlled laboratory atmospheric conditions (21–25 °C temperature and 40–60% relative humidity). The oxygen flow rates were selected based on WHO recommendations for supplemental oxygen delivery in neonates, infants, and older children (0.5–1, 1–2, and 1–4 LPM, respectively) [4] to enable direct comparison between the two oxygen delivery systems at matched flow rates.

### 2.3. Determination of Drug Delivery

Emitted dose determines the total drug that is emitted from the system at the end of the patient interface. A filter (Respirgard 303EU, Vyaire Medical, Basingstoke, UK) was used to capture aerosol at the end of the interface.

Tracheal dose was determined by placing a capture filter at the end of the trachea of a 3D-printed neonate head model. The model used was derived from a CT scan of the nasal passages of a fifteen-day-old male (gestation time of 31 weeks and 2 days and weighing 1.57 kg at the time of the CT scan). The head model and capture filter were connected to a breathing simulator (BRS 2100 breathing simulator, Copley Scientific, Nottingham, UK) which was set to 60 breaths per minute (BPM), a tidal volume (V_T_) of 25 mL, and an inspiratory:expiratory (I:E) ratio of 1:3. The respiratory rate and I:E ratio were based on published neonatal ventilation parameters [1,20], while the V_T_ of 25 mL was selected to align with established in vitro neonatal aerosol delivery methodology [17].

The mass of drug captured on the filters and residual drug on the T-piece was determined using UV–spectrophotometry (WPA Lightwave II, Biochrom Ltd., Cambridge, UK) at 276 nm and interpolation on a standard curve of salbutamol concentrations. In line with previous reports, drug recovery from the filter was shown to be 100 +/− 5% [21].

### 2.4. Effect of Nebulizer Refill Process on Circuit Pressure

Circuit pressure was measured to ensure that the operation of the A-VMN did not affect circuit dynamics. A pressure sensor (CITREX H5, IMT Analytics, Buchs, Switzerland) was placed between the trachea and the breathing simulator to characterize pressure at the lung level [14]. The pressure sensor has an accuracy of ±0.75% and a differential pressure measuring range of ±200 mbar, with measurements recorded at 0.5 s intervals enabling the detection of pressure changes of approximately 0.15 mbar. The observed small variations in peak circuit pressure (P_peak_) at each flow rate reflect the breathing simulator’s operational characteristics (60 BPM with very low tidal volume of 25 mL) rather than measurement noise, as the sensor resolution is sufficient to resolve clinically meaningful pressure differences. All pressure measurements were acquired using the device’s integrated FlowLab data logging software, and the device is calibrated annually in accordance with manufacturer specifications to ensure accuracy. Circuit pressure was measured at each supplemental gas flow rate tested. Measurements were taken at 0.5 s intervals for a period of 70 s. The initial pressure in the circuit was measured for the first 20 s (0–20 s). A simulated circuit break was induced for the following 20 s (20–40 s). This was followed by a 10 s recovery period (40–50 s) and a drug refill, where the A-VMN’s medication cup lid was opened for 10 s (50–60 s) before closing and a stabilization period (60–70 s).

### 2.5. Statistical Analysis

Results are expressed as a percentage of the nominal drug dose (mean ± standard deviation). One-way ANOVA was used to calculate the statistical significance of the results between the flow rates across both systems, while a paired Student *t*-test determined the significance of the results when comparing the oxygen concentrator and LFNO. Significance was considered at *p* ≤ 0.05. All testing was completed in independent triplicate (*n* = 3).

## 3. Results

### 3.1. Effects of Gas Flow Rate on Aerosol Delivery

#### 3.1.1. Emitted Dose Performance

For the oxygen concentrator, gas flow rate had a statistically significant impact on emitted dose (Table 1; *p* < 0.001). The largest emitted dose was achieved at 1.0 LPM, which was significantly higher than at 0.2 LPM (*p* < 0.001) and 5.0 LPM (*p* = 0.002).

For LFNO, gas flow rate also had a statistically significant impact on emitted dose (Table 2; *p* = 0.001). The lowest flow rate, 1.0 LPM, yielded the largest emitted dose, while the highest, 5.0 LPM, yielded the lowest. Significant pairwise differences were found between 1.0 and 5.0 LPM (*p* = 0.001) and between 4.0 and 5.0 LPM (*p* = 0.005).

**Figure 3:**
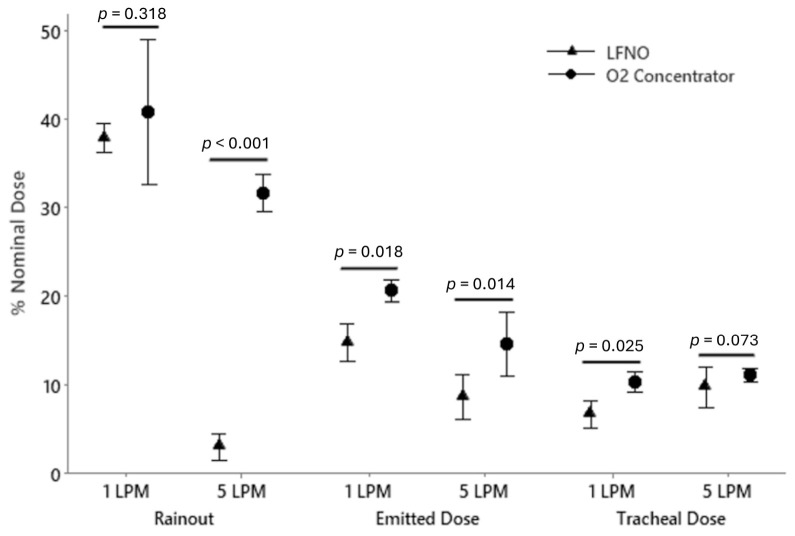
The percentage of drug delivered using the A-VMN with the Low-Flow Nasal Oxygen (LFNO) and Oxygen Concentrator (O_2_ Concentrator) at 1.0 LPM and 5.0 LPM. Error bars represent ± 1 standard deviation (*n* = 3).

At both comparable flow rates of 1.0 and 5.0 LPM, the oxygen concentrator produced a significantly higher emitted dose than LFNO (1.0 LPM: *p* = 0.018; 5.0 LPM: *p* = 0.014) (Figure 3).

#### 3.1.2. Rainout Characterization

With the oxygen concentrator, gas flow rate had a statistically significant impact on rainout within the T-piece (Table 1; *p* = 0.01). The lowest flow rate of 0.2 LPM generated the greatest rainout, which decreased progressively with increasing flow rate.

For LFNO, flow rate also had a statistically significant impact on rainout (Table 2; *p* < 0.001). Significant pairwise differences were found between 1.0 and 4.0 LPM (*p* < 0.001), 1.0 and 5.0 LPM (*p* < 0.001), and 4.0 and 5.0 LPM (*p* = 0.031).

At comparable flow rates, the oxygen concentrator produced substantially greater rainout than LFNO at 5.0 LPM (31.59 ± 0.87% vs. 2.90 ± 0.58%, *p* < 0.001), with no significant difference observed at 1.0 LPM (*p* = 0.318) (Figure 3).

#### 3.1.3. Tracheal Dose Efficiency

With the oxygen concentrator, gas flow rate had a statistically significant impact on tracheal dose (Table 1; *p* = 0.01). The highest tracheal dose was measured at 0.2 LPM, which was significantly higher than at 1.0 LPM (*p* = 0.016). There was no significant difference between 0.2 and 5.0 LPM.

For LFNO, gas flow rate also had a statistically significant impact on tracheal dose (Table 2; *p* = 0.018). The highest tracheal dose was recorded at 5.0 LPM, with the lowest at 1.0 LPM. A significant difference was found between 1.0 and 5.0 LPM only (*p* = 0.008).

At 1.0 LPM, the oxygen concentrator delivered a significantly higher tracheal dose than LFNO (10.24 ± 0.44% vs. 6.57 ± 0.60%, *p* = 0.025). At 5.0 LPM, the oxygen concentrator trended higher (11.01 ± 0.29% vs. 9.66 ± 1.53%), but this did not reach statistical significance (*p* = 0.073) (Figure 3).

### 3.2. Circuit Pressure Analysis

#### 3.2.1. Nebulizer Operation Safety

**Figure 4:**
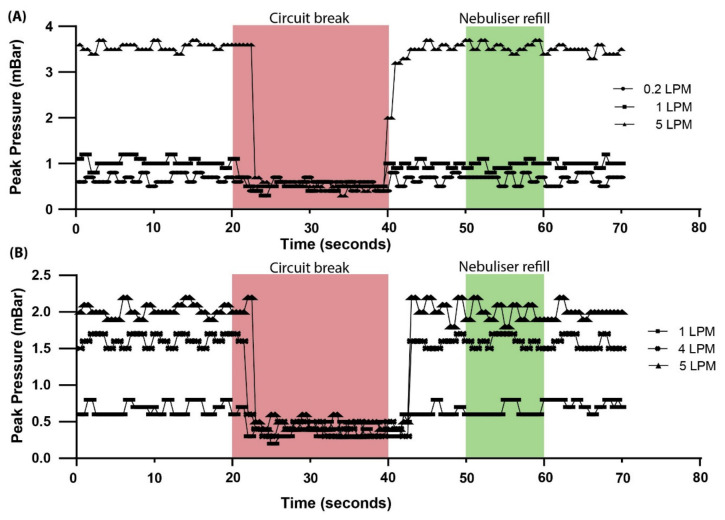
Peak circuit pressure (P_peak_) (mbar) during A-VMN operation, A-VMN refill, and intentional respiratory circuit break in both the (**A**) oxygen concentrator and (**B**) LFNO setup.

Figure 4 represents the peak circuit pressure measured in the (A) oxygen concentrator and (B) LFNO setups. An intentional break after 20 s caused P_peak_ to drop to approximately 0.3 mbar across all flow rates in both systems, from pre-break values of 3.6 mbar at 5.0 LPM, 1.0 mbar at 1.0 LPM, and 0.7 mbar at 0.2 LPM in the oxygen concentrator, and 2.0 mbar at 5.0 LPM, 1.7 mbar at 4.0 LPM, and 0.6 mbar at 1.0 LPM in the LFNO setup. P_peak_ recovered promptly on re-establishing the circuit.

#### 3.2.2. Refill Process Impact

No appreciable change in the P_peak_ during the nebulizer refill in both setups was noted. In the oxygen concentrator setup, P_peak_ ≈ 0.7 mbar at 0.2 LPM, 1.0 mbar at 1.0 LPM, and 3.5 mbar at 5.0 LPM. In the LFNO setup, P_peak_ ≈ 0.65 mbar at 1.0 LPM, 1.7 mbar at 4.0 LPM, and 2.0 mbar at 5.0 LPM. Signal variability is attributable to the small tidal volume and rapid breath pattern of the breathing simulator.

## 4. Discussion

This study demonstrates that supplemental gas flow rate and choice of oxygen delivery system significantly impact aerosol drug delivery efficiency. Use of the oxygen concentrator resulted in greater emitted and tracheal doses compared to LFNO across comparable flow conditions, most markedly at 1.0 LPM, although this did not always reach statistical significance. Operation and refill of the A-VMN had no adverse effect on circuit pressure in either system. To the best of the authors’ knowledge, this is the first study to characterize concurrent aerosol drug delivery during oxygen concentrator use in this clinical context. Our hypothesis that the absence of heated humidification in the oxygen concentrator circuit would result in greater aerosol delivery efficiency was supported.

### 4.1. Effect of Oxygen Flow Rate

At the clinically relevant flow rates assessed, the oxygen concentrator flow rate had a significant impact on the emitted dose, rainout, and tracheal dose. The lower emitted dose at 0.2 LPM is likely attributable to reduced convective velocity allowing gravitational sedimentation to become the dominant particle deposition mechanism within the circuit. At reduced flow rates, aerosol particles settle onto circuit surfaces due to gravitational forces rather than remaining suspended for delivery, a mechanism well-documented to be particularly significant for particles in the 1–5 µm size range at low inhalation flow rates [22]. Conversely, the lower emitted dose observed at 5.0 LPM is consistent with turbulence-driven aerosol impaction losses at elevated flow rates. Previous studies document that increased gas flow generates turbulence within the delivery circuit and nasal prongs, causing inertial impaction of aerosol particles ≥3 µm and deposition on circuit surfaces before reaching the patient [15]. This mechanism reduces the amount of aerosol available for delivery to the lower airway.

This finding aligns with published guidance recommending that aerosol therapy during nasal cannula oxygen delivery in children should not exceed 4 LPM to minimize turbulence-related losses [15].

In the LFNO setup, positioning the nebulizer at the dry side of the humidifier allows larger droplets to fall in the humidification chamber, reducing downstream rainout [23,24]. Flow rate also had a significant impact on the emitted dose, rainout, and tracheal dose. As with the oxygen concentrator, a flow rate of 5.0 LPM resulted in the lowest emitted dose (8.60 ± 1.02%), the highest tracheal dose (9.66 ± 1.53%), and the least rainout in the T-piece (2.90 ± 0.58%), consistent with previously reported delivery of 8.64% nominal dose at 4 LPM in a simulated pediatric patient receiving oxygen therapy via high-flow nasal cannula [25].

### 4.2. Effect of Supplemental Oxygen System

Comparing both systems, the oxygen concentrator delivered more aerosol in each test scenario, most likely attributable to the absence of heated humidification in the oxygen concentrator circuit. Alcoforado et al. (2019) demonstrated in adults that unheated gas delivery yields higher aerosol lung deposition than actively heated humidified gas, a mechanistic finding that applies regardless of patient age. The underlying principle that heated, humidified gas promotes condensational loss in the delivery circuit is independent of flow rate and patient size [26]. This is further supported by recent in vitro infant data showing that reducing humidity-related condensation within a HFNO breathing circuit significantly improves the delivered aerosol dose [27]. In the LFNO system, carrier gas heated to approximately 37 °C promotes condensation and deposition within the circuit, reducing the quantity available for delivery. In the unheated concentrator circuit, cooler, drier gas reduces condensational loss in transit, yielding a higher emitted dose at the patient interface. This is consistent with the substantially greater T-piece rainout in the concentrator arm, suggesting that condensation occurred closer to the patient interface. However, using the oxygen concentrator resulted in the greatest amount of rainout in the T-piece compared to LFNO at both 1.0 LPM (40.77 ± 3.30% vs. 37.87 ± 0.67%, *p* = 0.318) and 5.0 LPM (31.59 ± 0.87% vs. 2.90 ± 0.58%, *p* < 0.001).

Tracheal doses here (6.57–11.98%) exceed those reported by Réminiac et al. [17] (3.3–4.2%) in an in vitro infant model. Methodological differences are likely to account for this. The present study used a 3D-printed model with characteristically narrow premature neonatal nasopharyngeal geometry, derived from specific CT data, whereas Réminiac et al. [17] employed the standardized SAINT model [28] representing a 9-month-old toddler with larger nasal passages where greater nasal filtration would be expected to reduce tracheal dose. Differences in tidal volume and breathing parameters between studies are also likely contributors. The calculated delivered mg/kg dose for both delivery systems exceeds the weight-based dose of 0.15 mg/kg per nebulization reported in clinical trials of nebulized salbutamol in infants, indicating that the system achieves bronchodilator deposition at therapeutically relevant doses [29]. These values nonetheless compare favorably with DiBlasi et al. [30], who reported neonatal inhaled lung doses of only 0.2–0.8% during HFNO therapy.

### 4.3. Effect of Nebulizer Refill Process on Circuit Pressures

Concurrent aerosol therapy via the A-VMN did not affect the circuit pressure during oxygen therapy or nebulizer refill, consistent with previous reports [14,31,32]. This was observed across both supplemental oxygen delivery systems and all flow rates tested, with P_peak_ recovering promptly following the intentional circuit break. A decrease in circuit pressure can cause airway derecruitment or atelectasis in an already compromised patient [20].

There are several limitations to this study. As in vitro research, results may not fully reflect drug delivery in patients with respiratory disease [33,34]. A single head model was used and model choice is known to impact aerosol delivery [35]. Findings are specific to the Aerogen Solo with A-VMN and cannot be extrapolated to other vibrating mesh nebulizer systems. The head model was tested in an upright rather than supine orientation, which does not reflect typical neonate clinical positioning, and head orientation may influence aerosol deposition patterns [30].

The tidal volume of 25 mL aligns with the established in vitro methodology of Réminiac et al. [17] and similar infant aerosol studies, but exceeds the weight-matched physiological range for a 1.57 kg premature neonate (approximately 6–10 mL) [20]. As tidal volume positively affects aerosol delivery in small airway models [34], absolute tracheal dose values are likely overestimated relative to in vivo conditions. Importantly, both supplemental oxygen delivery systems were tested under identical conditions, so between-system comparisons are unaffected by this choice of tidal volume.

Future studies should examine the effect of heated humidification, explore alternative patient interfaces, and extend findings to broader pediatric age groups, with in vivo validation of significant clinical value. Recent evidence demonstrating the feasibility of A-VMN-based drug delivery in premature neonates [36] further supports the translational relevance of these findings.

## 5. Conclusions

This study compared concurrent aerosol and supplemental oxygen delivery to a simulated neonate model using an A-VMN with an oxygen concentrator and LFNO therapy. Both the gas flow rate applied and the oxygen supplementation system used had a significant impact on the amount of aerosol delivered. The safety of concurrent A-VMN use is supported by the absence of any clinically meaningful change in circuit pressure during nebulizer operation or refill in either system. Based on the metrics tested, the oxygen concentrator was the most effective supplementary oxygen delivery system for aerosol delivery. These findings are novel and have direct implications for clinical practice in settings where oxygen concentrators are used.

## Figures and Tables

**Table 1:** Aerosol drug delivery via A-VMN with an oxygen concentrator expressed as a % of the nominal dose. All results are expressed as the mean ± SD.

Flow Rate (LPM)	Rainout (%)	Emitted Dose (%)	Tracheal Dose (%)
0.2	56.81 ± 11.08	13.04 ± 1.91	11.98 ± 0.60
1.0	40.77 ± 3.30	20.58 ± 0.50	10.24 ± 0.44
5.0	31.59 ± 0.87	14.49 ± 1.45	11.01 ± 0.29
One-way ANOVA *p*-value *	0.01	<0.001	0.01

* *p*-values represent one-way ANOVA across all flow rates for each outcome measure.

**Table 2:** Aerosol drug delivery via A-VMN and low-flow nasal oxygen expressed as a % of the nominal dose. All results are expressed as the mean ± SD.

Flow Rate (LPM)	Rainout (%)	Emitted Dose (%)	Tracheal Dose (%)
1.0	37.87 ± 0.67	14.69 ± 0.89	6.57 ± 0.60
4.0	6.47 ± 1.60	14.40 ± 1.49	8.50 ± 1.17
5.0	2.90 ± 0.58	8.60 ± 1.02	9.66 ± 1.53
One-way ANOVA *p*-value *	<0.001	0.001	0.018

* *p*-values represent one-way ANOVA across all flow rates for each outcome measure.

## Data Availability

The raw data supporting the conclusions of this article will be made available by the authors upon reasonable request.

## Abbreviations

The following abbreviations are used in this manuscript:

A-VMN	Aerogen vibrating mesh nebulizer
BPM	Breaths per minute
CT	Computed tomography
HFNO	High-flow nasal oxygen
I:E	Inspiratory:expiratory (ratio)
LFNO	Low-flow nasal oxygen
LPM	Liters per minute
P_peak_	Peak circuit pressure
SAINT	Sophia Anatomical Infant Nose-Throat
UV	Ultraviolet
VMD	Volumetric mean diameter
V_T_	Tidal volume
WHO	World Health Organization
